# Supplementary material for: Prevalence of Dichelobacter nodosus and Ovine Footrot in German Sheep Flocks
Source: Animals (Basel). 2021 Apr 12;11(4):1102. doi: 10.3390/ani11041102 (PMC8069605; doi:10.3390/ani11041102)
Supplement: Supplementary file 1 [file animals-11-01102-s001.zip › Table S1_Revised.docx]

**Table S1.** Numbers and frequencies of real-time PCR results of 9,243 swab samples

| **Real-Time PCR Result** | **Number of Sheep** | **Frequency (%)** |
| --- | --- | --- |
| Free of *D. nodosus* | 5275 | 57.07 |
| Benign *D. nodosus (aprB2+)* | 188 | 2.03 |
| Benign and virulent *D. nodosus (aprB2+/aprV2+)* | 195 | 2.11 |
| Virulent *D. nodosus (aprV2+)* | 3585 | 38.79 |
| Total | 9243 | 100 |
