# Supplementary material for: Prevalence of Dichelobacter nodosus and Ovine Footrot in German Sheep Flocks
Source: Animals (Basel). 2021 Apr 12;11(4):1102. doi: 10.3390/ani11041102 (PMC8069605; doi:10.3390/ani11041102)
Supplement: Supplementary file 1 [file animals-11-01102-s001.zip › Table S2_Revised.docx]

**Table S2.** Survey of previous studies using PCR testing on *D. nodosus*

| **Reference** | **Country** | **Sampling Scheme** | **Number of Flocks/Abattoirs** | **Detection Method** | **Number of Inspected Animals** | **Number of Samples Tested Using PCR** | **Differentiation of Benign and Virulent *D. nodosus*** | **Scoring System** | **Sampling Technique** | **Prevalence** |
| --- | --- | --- | --- | --- | --- | --- | --- | --- | --- | --- |
| **Ardüser et al., 2020 [14]** | Switzerland | On farm visit | 142 | Real-time PCR | 2920 | 2920 | Yes | / | Single four-feet swab samples | Virulent *D. nodosus:* 16.9%  Benign *D. nodosus:* 6.3% |
| **Kraft et al., 2020 [17]** | Germany | On farm visit | 30 | Real-time PCR | 897 | 897 | Yes | 0–5 | Single four-feet swab samples | Virulent *D. nodosus:* 6 flocks  Benign *D. nodosus:* 6 flocks |
| **König et al., 2011 [19]** | Sweden | Abattoir | Unknown/6 | 16S real-time PCR  + culture | 471 | 29 | No | 0–5 | Single one-feet swab samples | Footrot in 5.8% of slaughter lambs |
| **Maboni et al., 2016 [21]** | United Kingdom | Abattoir | Unknown/1 | Real-time PCR | unknown | 241 | Yes | Healthy,  mild ID, moderate-to-severe ID, footrot | Interdigital post-slaughter biopsies | *D. nodosus*: 73.03%  Virulent *D. nodosus*: 67.63%  Benign *D. nodosus*: 7.05% |
| **The surveillance programme for footrot in Norway, 2019 [24]** | Norway | Abattoir | 116/6 | Multiplex 16S real-time PCR | 118.000 | 178 | Yes | 0-5 | Single one-feet swab samples and two-feet swab samples (sheep with clinical signs) | Virulent *D. nodosus* was detected in two animals in one sheep flock |
| **New South Wales Animal Health Surveillance, 2018 [26]** | Australia, New South Wales | On farm visit, saleyards | Unknown | Unknown | Unknown | Unknown | Yes | 0-5 | Unknown | North Coast, Northern Tablelands, Hunter, Greater Syndey: 0%  Central West: 0.24%  Western: 0.26%  South East: 0.34%  Riverina: 0.59%  Central Tablelands: 0.63%  Murray: 1.31% |
