# Supplementary figures and images for: Prevalence of Dichelobacter nodosus and Ovine Footrot in German Sheep Flocks
Source: Animals (Basel). 2021 Apr 12;11(4):1102. doi: 10.3390/ani11041102 (PMC8069605; doi:10.3390/ani11041102)

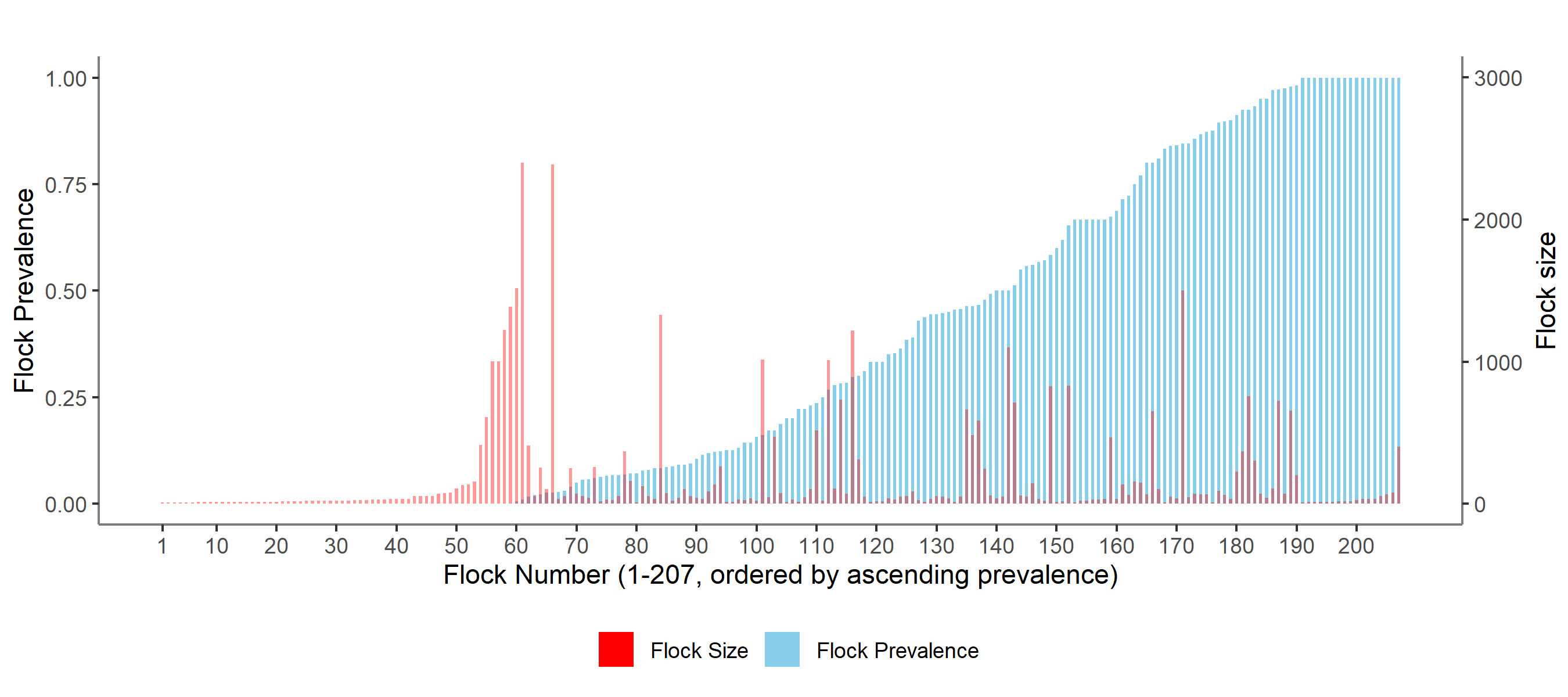

Supplement: Supplementary file 1 [file animals-11-01102-s001.zip › Figure S1.tiff]

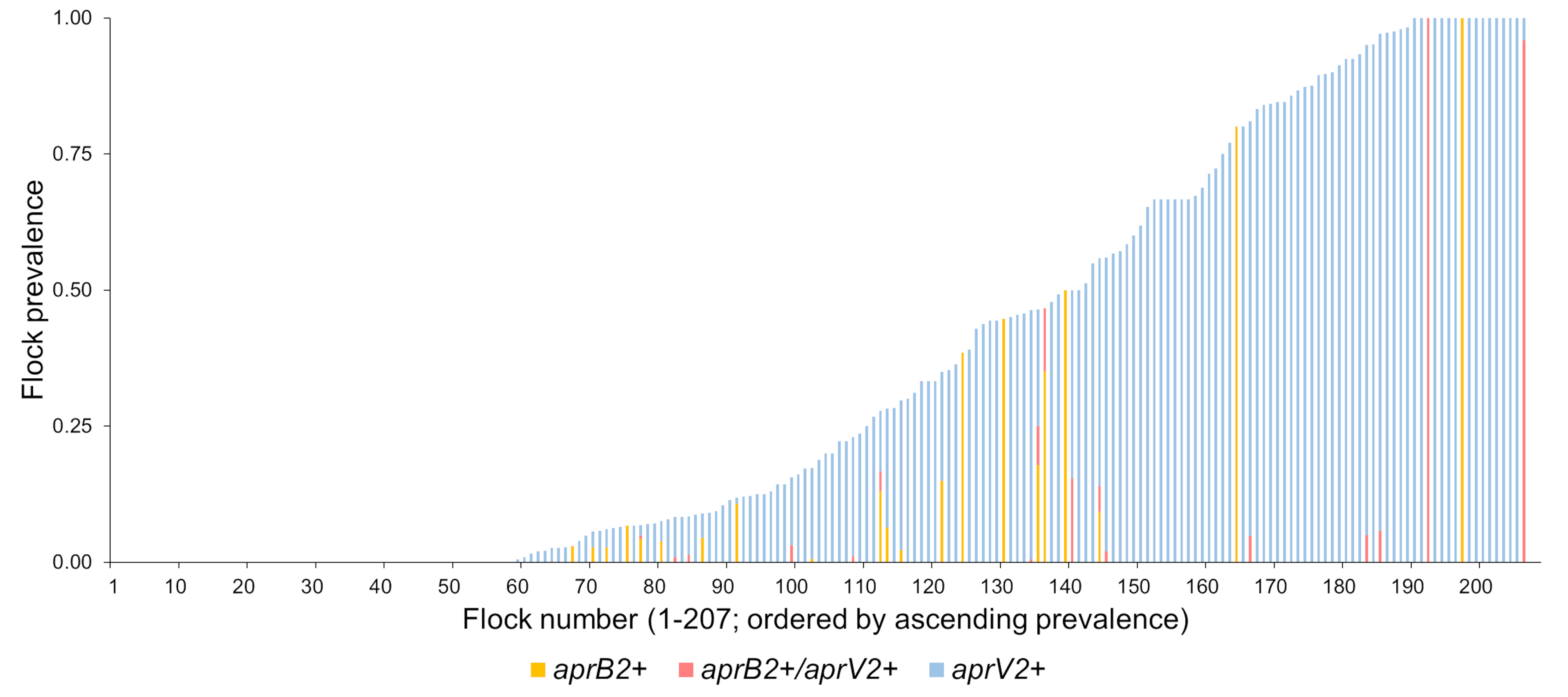

Supplement: Supplementary file 1 [file animals-11-01102-s001.zip › Figure S2_Flock prevalence virulent benigne.tiff]
